# Few-cycle Regime Atomic Force Microscopy

**DOI:** 10.1038/s41598-019-49104-1

**Published:** 2019-09-03

**Authors:** Enrique A. López-Guerra, Suhas Somnath, Santiago D. Solares, Stephen Jesse, Gabriele Ferrini

**Affiliations:** 1 Department of Mechanical and Aerospace Engineering, The George Washington University, Washington, DC 20052 USA; 2 Department of Civil and Environmental Engineering, The George Washington University, Washington, DC 20052 USA; 3 National Center for Computational Sciences, Oak Ridge National Laboratory, Oak Ridge, Tennessee 37831 USA; 4 Center for Nanophase Materials Sciences, Oak Ridge National Laboratory, Oak Ridge, Tennessee 37831 USA; 5 Institute for Functional Imaging of Materials, Oak Ridge National Laboratory, Oak Ridge, Tennessee 37831 USA; 6 Interdisciplinary Laboratories for Advanced Materials Physics, Università Cattolica del Sacro Cuore, I-25121 Brescia, Italy; 7 Dipartimento di Matematica e Fisica, Università Cattolica del Sacro Cuore, I-25121 Brescia, Italy; 8 Present Address: Park Systems Inc., Santa Clara, CA 95054 USA

**Keywords:** Characterization and analytical techniques, Imaging techniques

## Abstract

Traditionally, dynamic atomic force microscopy (AFM) techniques are based on the analysis of the quasi-steady state response of the cantilever deflection in terms of Fourier analysis. Here we describe a technique that instead exploits the often disregarded transient response of the cantilever through a relatively modern mathematical tool, which has caused important developments in several scientific fields but that is still quite unknown in the AFM context: the wavelet analysis. This tool allows us to localize the time-varying spectral composition of the initial oscillations of the cantilever deflection when an impulsive excitation is given (as in the band excitation method), a mode that we call the *few-cycle regime*. We show that this regime encodes very meaningful information about the tip-sample interaction in a unique and extremely sensitive manner. We exploit this high sensitivity to gain detailed insight into multiple physical parameters that perturb the dynamics of the AFM probe, such as the tip radius, Hamaker constant, sample’s elastic modulus and height of an adsorbed water layer. We validate these findings with experimental evidence and computational simulations and show a feasible path towards the simultaneous retrieval of multiple physical parameters.

## Introduction

Atomic force microscopy has established itself as an indispensable tool in nanoscience^1^. Following simple contact topography measurements, many techniques have been created to image atomic structures and probe mechanical, electrical, magnetic, and biophysical phenomena at the nanoscale^2,3^. Amplitude/Frequency modulation^4^ and force spectroscopy^5^ are the most widely used techniques since the variation of a single averaged parameter (amplitude, frequency, deflection) carries information on the tip-sample interaction, easing data interpretation. While periodic tip-sample excitation has permeated AFM modes from its inception^6^, comparatively less attention has been devoted to techniques where single or few tip-sample interactions are used to characterize the sample^7,8^. In analogy with developments in ultrafast optics where short laser pulses are used to excite delta-like mechanical pulses in various kinds of nanostructures^9^, tip induced mechanical pulses could similarly be used to open new detection channels in a different part of the mechanical spectrum of a nanostructure.

The aim of the present work is to characterize the few-cycle regime atomic force microscopy (FR-AFM) and show its capability for measuring multiple physical parameters (e.g., sample’s mechanical properties, adhesion of molecular layers, surface structuring) in a very sensitive manner. In this FR-AFM technique, the time response of an interacting cantilever under impulsive excitation will be investigated with a mathematical tool well suited for the study of non-stationary signals: the wavelet transform. This analysis will allow us to perform a time localization of the spectral components of the transient response of the cantilever in the few-cycle regime. As a result, we will be able to measure the instant amplitude, frequency and relative phase of the fundamental mode^10^. To accomplish this task, the following three requirements must be satisfied: (i) we need an instrument capable of delivering customizable, large bandwidth, pulse-like excitations to a cantilever; (ii) a method of monitoring the deflection and digitizing all the responses of interest with sufficient resolution; (iii) a robust analysis protocol capable of unraveling and processing the data.

## Few-Cycle Regime Atomic Force Spectroscopy

### Preliminaries

Before introducing the few-cycle regime atomic force spectroscopy, it is useful to discuss the principles of already established methods using the band excitation scheme^11^. Band excitation methods start from the observation that only regions around resonances contain information of practical interest, so the cantilever is periodically excited over a frequency band near a resonance, by sweeping the cantilever excitation frequency. The excitation and response signals are usually analyzed using the Fourier transform to retrieve the transfer function of the interacting cantilever at each pixel of a scanned area^12^. The amplitude-frequency and the phase-frequency curves are fitted with a simple harmonic oscillator (SHO) model. The fit allows to retrieve the three fundamental parameters, i.e. the resonance frequency, the oscillation amplitude and the Q factor. This analysis conveys the fundamental information on the cantilever dynamics, however it does not capture the evolution of the amplitude and phase of the interacting cantilever on the interaction time-scale. In fact, Fourier analysis has a definite meaning only for quasi-stationary signals. When the interaction develops over few cycles of the cantilever oscillation, amplitude, phase and frequency are time-dependent. Their instantaneous values (subject to the uncertainty principle limits), encode the information on the tip-sample interaction.

To explore the dynamics of the cantilever subjected to impact phenomena, experiments have been conducted using the jump-to-contact (JTC) transition in conjunction with the thermal oscillation regime, which can be considered an extreme form of broad band excitation with a random phase^13^. The evolution of the excited modes during the tip impact after a JTC transition on a graphite surface has been reconstructed using an analysis technique based on wavelet transforms^14,15^. The instantaneous total force acting on the tip during a single impact and the energy dissipation has been measured^8,16^. These preliminary measurements have shown a promising pathway for the development of an atomic force spectroscopy dynamic method based on few oscillation cycles of the cantilever. However, the use of the JTC transition does not allow control of the force applied to the cantilever and the driving forces are unknown, depending on the surface forces between tip and sample that may vary unpredictably from experiment to experiment. To circumvent the issues associated to the JTC transition, a method based on impulsive (pulse-like) cantilever excitation has been theoretically proposed^17^.

Here, we implement experimentally the idea by exciting the cantilever with an excitation pulse whose amplitude and phase are known. The amplitude and phase of the cantilever response after the tip has interacted with the sample is characterized by a wavelet cross-correlation technique. In this way it is possible to quantify the phase difference between the response of the cantilever and the exciting driver force, yielding instantaneous information on the frequency, phase and amplitude shift as a function of time (details will be provided in the following sections).

### Implementation of the few-cycle regime atomic force spectroscopy

**Figure 1:**
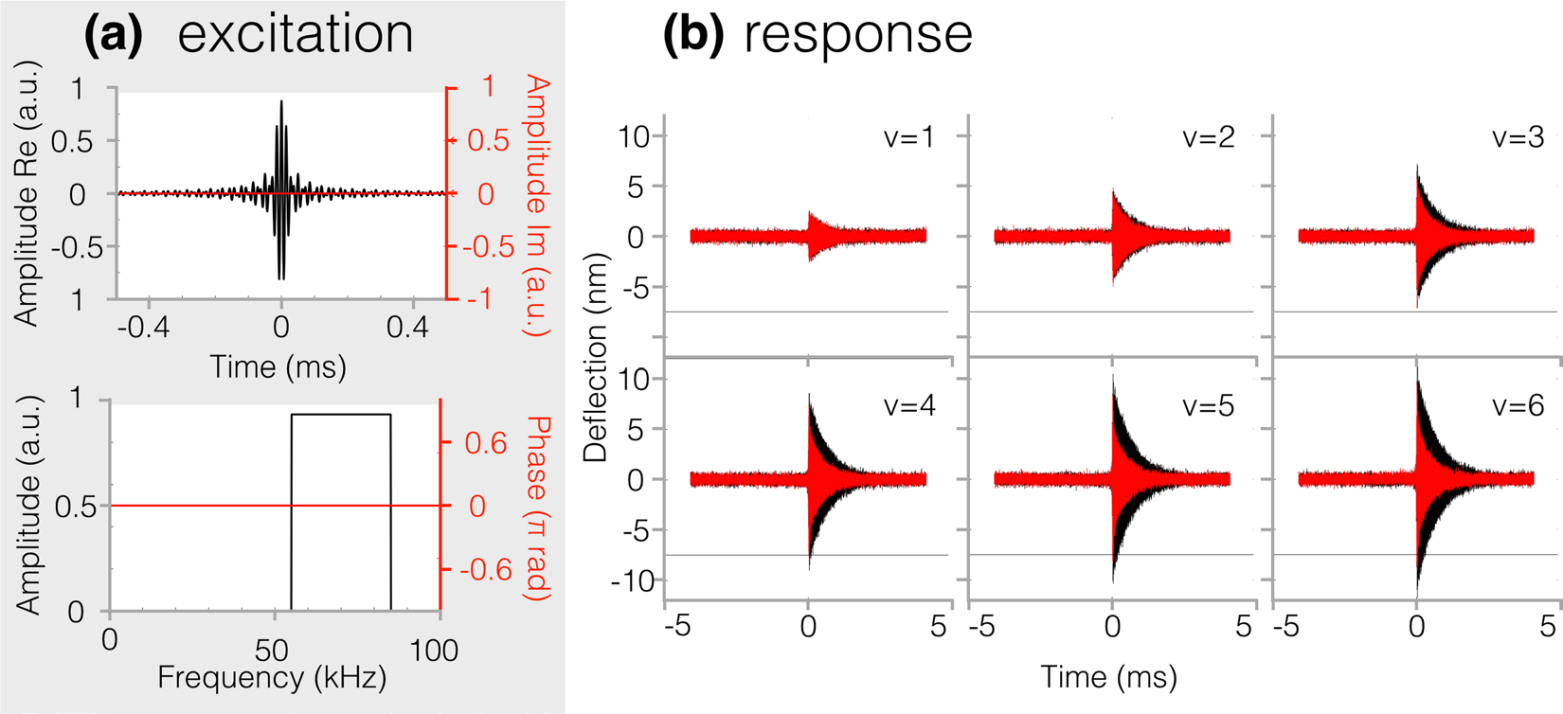
Scheme of the implementation of the few-cycle regime atomic force microscopy. Panel (a) shows the shape of the cantilever excitation signal in the time domain (a modulated sinc excitation) and in the frequency domain (band excitation). Panel (b) shows the tip-sample deflection time dynamics (as a result of the excitation shown in panel (a)) as a function of varying excitation intensity (from v = 1 to v = 6 in increasing order). The black traces are the response of the free cantilever, the red traces are the response of the interacting cantilever, with a tip rest position at 7.5 nm from the surface. The gray horizontal line marks the 7.5 nm deflection amplitude to help visualize when the cantilever interacts with the surface.

In this work we drive the cantilever photothermally with a pulsed excitation (applied with a digital signal generator) whose frequency band is centered on the first cantilever resonance (band limited pulse) with a constant spectral phase set to zero (see Fig. 1(a)). In the time domain, the excitation envelope resembles a sinc-like function with a duration inversely proportional to the excitation bandwidth (see Fig. 1(a)). The maximum of the excitation pulse is defined as the zero of the time axis.

The cantilever is approached to the sample at a specified equilibrium distance (details on determining this distance given in Supporting Material) and excited with the signal described in Fig. 1(a) with six distinct values of excitation amplitude. For each excitation amplitude the cantilever’s deflection response caused by the pulsed sinc-like excitation is read through the photodiode and appropriately sampled to retrieve the full temporal evolution of the interacting tip up to the higher flexural modes of interest (see red traces in Fig. 1(b)). Additionally, a free-response (i.e., absence of tip-sample interactions) of the tip’s deflection is also recorded for future reference (black traces in Fig. 1(b)). When the cantilever is free, the positive and negative deflections induced by the impulsive excitation are symmetric and centered on zero. When the probe is interacting, the attractive and repulsive tip-sample interactions cause a drop in the amplitude response. This is clear by comparing the interacting traces (red traces) and free-response traces (black traces) in Fig. 1(b). For the three lower excitation amplitudes (upper panel in Fig. 1(b)), the amplitude drop is ruled by attractive interactions only. Whereas for the higher excitation amplitudes (lower panel in Fig. 1(b)), the probe dynamics are affected by both attractive and repulsive forces, which cause a further reduction in the amplitude. The horizontal line in Fig. 1(b) marks a deflection equal to the tip-sample equilibrium distance.

## Wavelet Analysis

### Wavelet spectrogram

**Figure 2:**
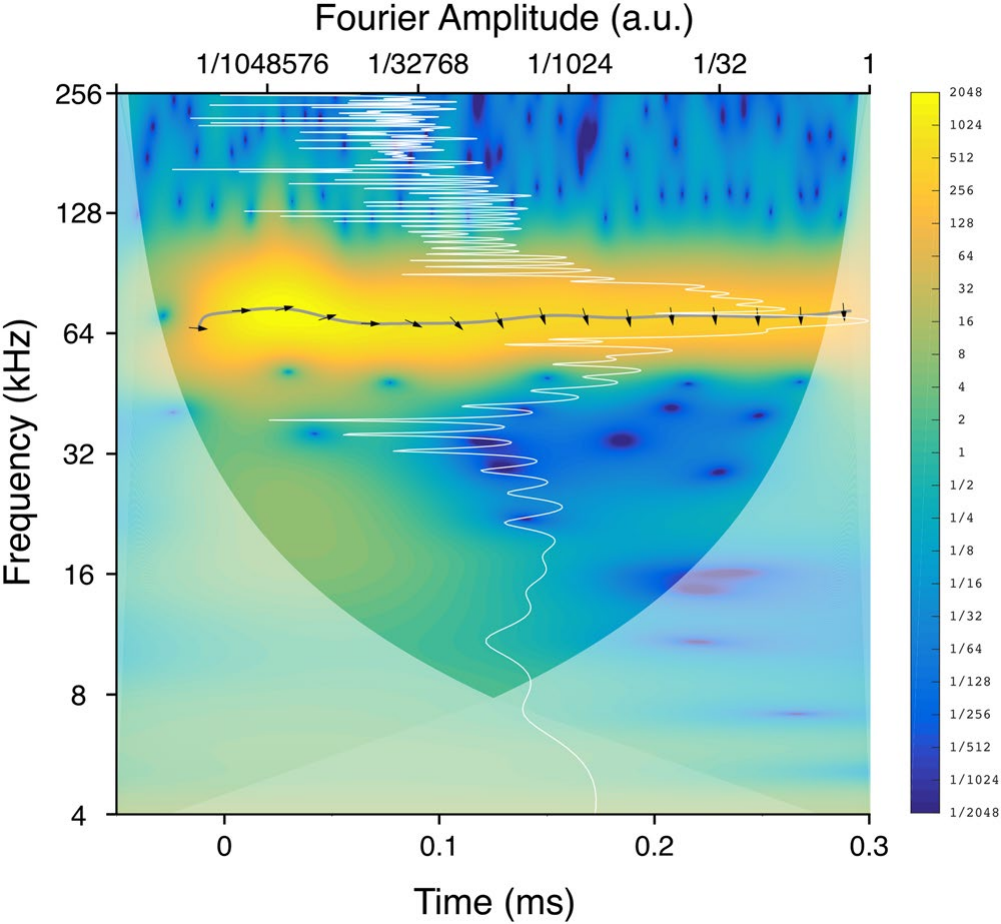
Wavelet transform of the cantilever’s deflection (the *signal*) that is interacting with a highly oriented pyrolytic graphite (HOPG) sample when the band excitation described in Fig. 1(a) is applied to it. The ambient humidity is controlled and reduced to a nominal zero. The cantilever rest position is at 7.5 nm from the surface. The colors in the spectrogram represent the squared modulus (magnitude) of the wavelet transform. The frequency scale, the Fourier amplitude scale and the color scale are logarithmic base 2 (octaves) while the time scale is linear. The Fourier transform (white line, left and top axis) is superposed on the wavelet transform. The gray line under the arrows is the instantaneous frequency associated with the first flexural mode during the cantilever excitation; the arrows’ rotation shows the instantaneous phase shift between signal and reference. The reference is taken to be the cantilever’s deflection when subject to the same band excitation while located far from the sample (in the absence of tip-sample interactions). Arrows pointing right: signal in phase with reference, phase shift = 0; left: signal in anti-phase with reference, phase shift = *π*; up: signal leading reference by *π*/2, phase shift = *π*/2; down: signal lagging reference by *π*/2, phase shift = −*π*/2.

We introduce here the analysis of the few-cycle regime in the experimental traces (i.e. the cantilever’s deflection, from now on referred to as *the signal*) through wavelet transforms (WT). Each signal is analyzed by a WT (mathematical details provided in Supporting Information). The amplitude of the WT can be represented in a 2D spectrogram (see Fig. 2) where the spectral components of the signal are localized in time and frequency.

The instantaneous frequency, is represented as a path superposed on the wavelet spectrogram in the frequency-time plane. Technically the instantaneous frequency is defined by the ridges of a normalized spectrogram^18^ and is represented in Fig. 2 as a gray line. In our case the instantaneous frequency represents the time evolution of the frequency of the first mode of the cantilever. Due to the impulsive excitation and because of the tip-sample interaction, the frequency of the first mode of the cantilever is modified, oscillating around the frequency of the undisturbed mode.

Using a cross wavelet transform we calculate the instantaneous phase difference between the signal and a reference trace. The reference trace is that of a free cantilever, a cantilever where the tip is not interacting with the sample but oscillating in the same ambient medium as the signal. Although a phase difference is available at every point of the time-frequency plane where signal and reference are superposed, we show only the phase difference along the instantaneous frequency path (the gray line in Fig. 2 to limit the quantity of information to analyze. On the gray line the arrows represent the instantaneous phase through their rotation according to the convention reported in the figure’s caption. In this figure it is possible to follow the evolution of the magnitudes of the *signal dynamical variables* (instantaneous frequency, amplitude and phase difference) as a function of time, forming the *dynamical trajectories*. In Fig. 2 we also show, for reference purposes, the Fourier transform represented as a white line over the wavelet spectrogram. The Fourier transform represents the limiting case in which the spectral components of the signal are highly localized in frequency at the expense of poor localization in time, following Heisenberg’s uncertainty principle^18^.

### 3D metric space

**Figure 3:**
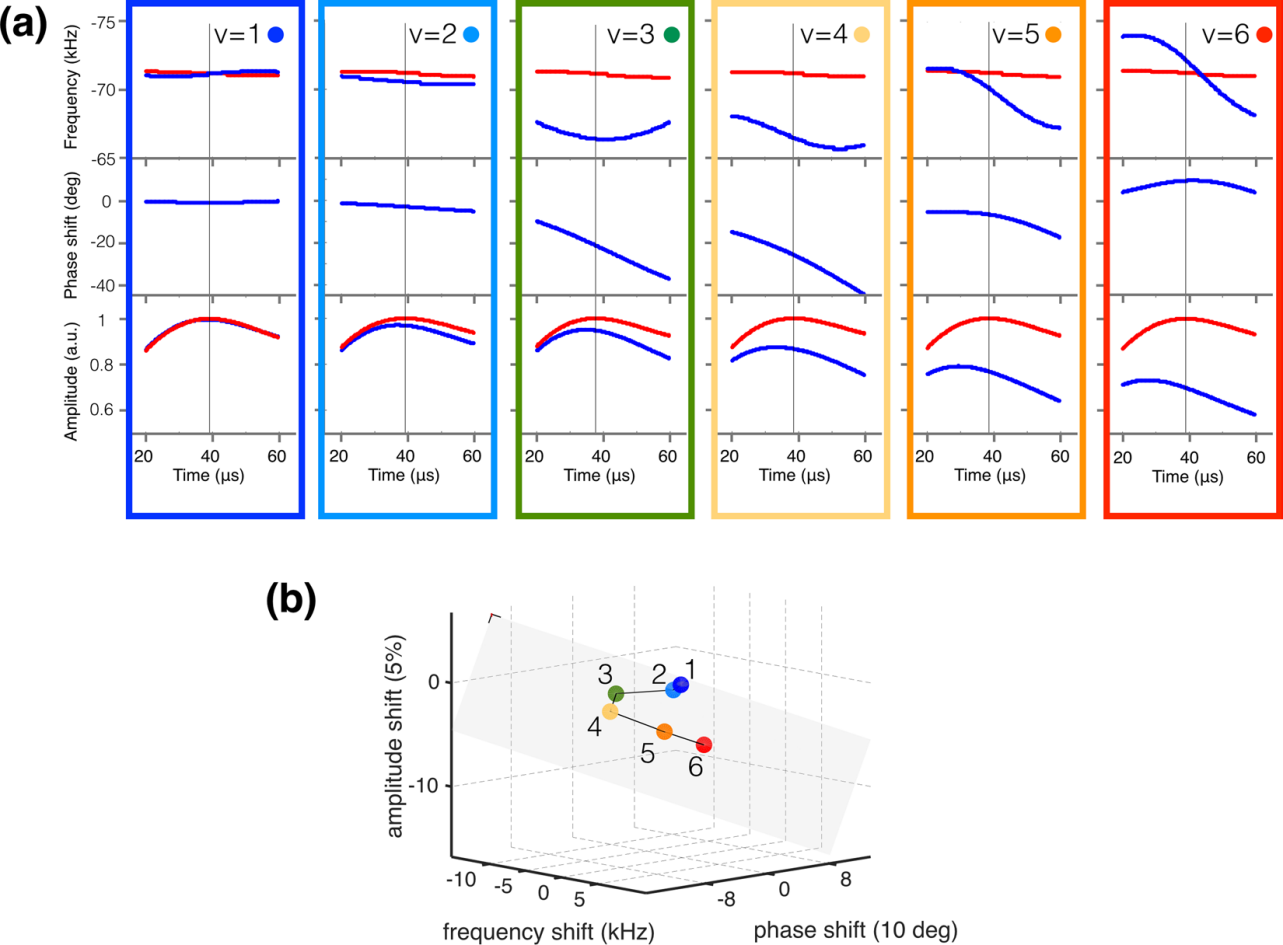
Representation of the steps involved in extracting the *metric trajectories* that are localized in the instantaneous frequency-amplitude-phase 3D space (the *ifap 3D space*). (**a**) Instantaneous time-varying values of frequency, phase shifts and amplitude, top to bottom, comprising the *dynamical trajectories*. In all plots the red traces correspond to the trajectories describing the evolution of amplitude and frequency for the free cantilever (no tip-sample interaction), the blue traces for the interacting cantilever. Since in this context the absolute phases do not have a physical meaning, only the phase shifts of the interacting cantilever with respect to the free cantilever are shown. The external band excitation amplitude increases linearly from left to right, from v = 1 to v = 6. These trajectories are measured along the instantaneous frequency trajectory in the time-frequency plane of the wavelet transform (see gray line in Fig. 2). (**b**) *Metric trajectory* in the ifap 3D space. We have color-coded and numbered each distinct interacting case in the upper plots and associated it with a unique point in the lower plot. To do so, we define a metric as the difference between the dynamical variables of the interacting cantilever and those corresponding to the free cantilever by observing their values at the time when the maximum free amplitude occurs (vertical light gray line). By repeating this process for a family of experiments performed at the same equilibrium position (7.5 nm in this case) and varying excitation amplitude, we generate a *metric trajectory* that is evidenced by the black line connecting the metric points. This line expands from a place near the origin (lowest excitation amplitude and quasi-free cantilever) to points further from the origin as the excitation intensity increases (interacting cantilever). All the points lay almost exactly on a plane, represented in shaded gray with its normal pointing to the observer.

Each signal is characterized by three *tunable parameters*: the cantilever resting position, the intensity of the excitation, and the ambient relative humidity (RH). In Fig. 3(a) we show the time-varying values of amplitude, frequency and phase for the *signal* and *reference* for a set of experiments where all tunable parameters are kept constant, but just the excitation intensity is varied. These time-varying values of amplitude, frequency and phase (i.e. the dynamical trajectories) are calculated along the instantaneous frequency path (gray line in Fig. 2). To observe how dynamical trajectories are affected by the different tunable parameters in an intuitive manner we construct a *metric* that summarizes the trajectory evolution in few parameters. The metric is constructed as follows. We fix a time reference choosing the instant at which the *free* cantilever (reference) amplitude is maximum. Then, the instantaneous values of amplitude, phase difference and frequency are evaluated at that time reference. As a result, a set of three values (that we call *metric values*) specifying frequency shift, phase shift and amplitude shift are obtained for each combination of the tunable parameters.

If we continue generating these metric values for different experiments where a specific tunable parameter is modified (while the rest are kept constant), we end up with a set of points in the *3D metric space* as the one in Fig. 3(b). In this case we have modified only the excitation intensity. We regard the excitation intensity as a ‘special’ tunable parameter because it directly influences the degree of the tip-sample interaction. Therefore, we connect lines through the metric values where only this parameter is modified while the other two (room humidity and cantilever’s rest position) are constant. These connected lines are going to be referred as the metric trajectories.

It is important to note that the points in these graphs are not scattered in the volume but lay almost exactly on a plane. This indicates that a linear relationship exist between the three variables that determine the points’ positions: once two are chosen the third is determined.

## Discussion

The characteristic bending of the metric trajectory seen in Fig. 3(b) is a general feature. Initially the trajectory grows in an outward direction very nearly along a line starting from zero (linear region). In the linear region the frequency shifts and phase shifts are negative, which is typical of attractive interactions due to the tip attraction by the surface via van der Waals forces. As the excitation intensity increases in the linear region, the tip interacts with the attractive forces for a longer time, red-shifting proportionally the cantilever oscillation frequency and decreasing the phase shift. On a further increase of excitation intensity, the tip starts to interact with the surface and repulsive forces come into play. Due to the repulsive interactions the trajectory bends towards positive frequency and phase shifts. The amplitude decreases monotonically upon increasing of the interaction, irrespective of this being attractive or repulsive. It is interesting to note that the points forming the metric trajectory develop (almost exactly) on a plane. The characteristic shape of this bending encodes information on the external parameters, that ultimately regulate the dynamical trajectories and consequently the metric trajectories.

To rationalize these metric trajectories and gain information about physical parameters that influence the cantilever dynamics (e.g., the mechanical properties of the surface, the characteristics of the interface as the water layer, the medium in which the cantilever oscillates) we simulate the tip trajectory using a numerical model. The model output is analyzed through the wavelet analysis with the same method as the experimental traces (i.e., processes described in Figs 2 and 3). The numerical model is chosen to be as simple as possible provided it captures the principal physical mechanisms responsible for the shape of the metric trajectories. The model is based on the contribution from the first three eigenmodes of the cantilever (see details in Methods section). In particular, the contribution of capillary force is taken into account by considering the presence of a water layer of height *h* on the sample surface. The presence of a wetting layer is fundamental to obtain a qualitative agreement between simulations and experiments. The Hamaker constant and the tip radius are the other important parameters to adjust in the simulations to retrieve similar metric trajectories as in the experiment. A general agreement between experimental and simulated metric trajectories must be obtained for every excitation intensity by choosing the appropriate simulation parameters for a given displacement and relative humidity.

**Figure 4:**
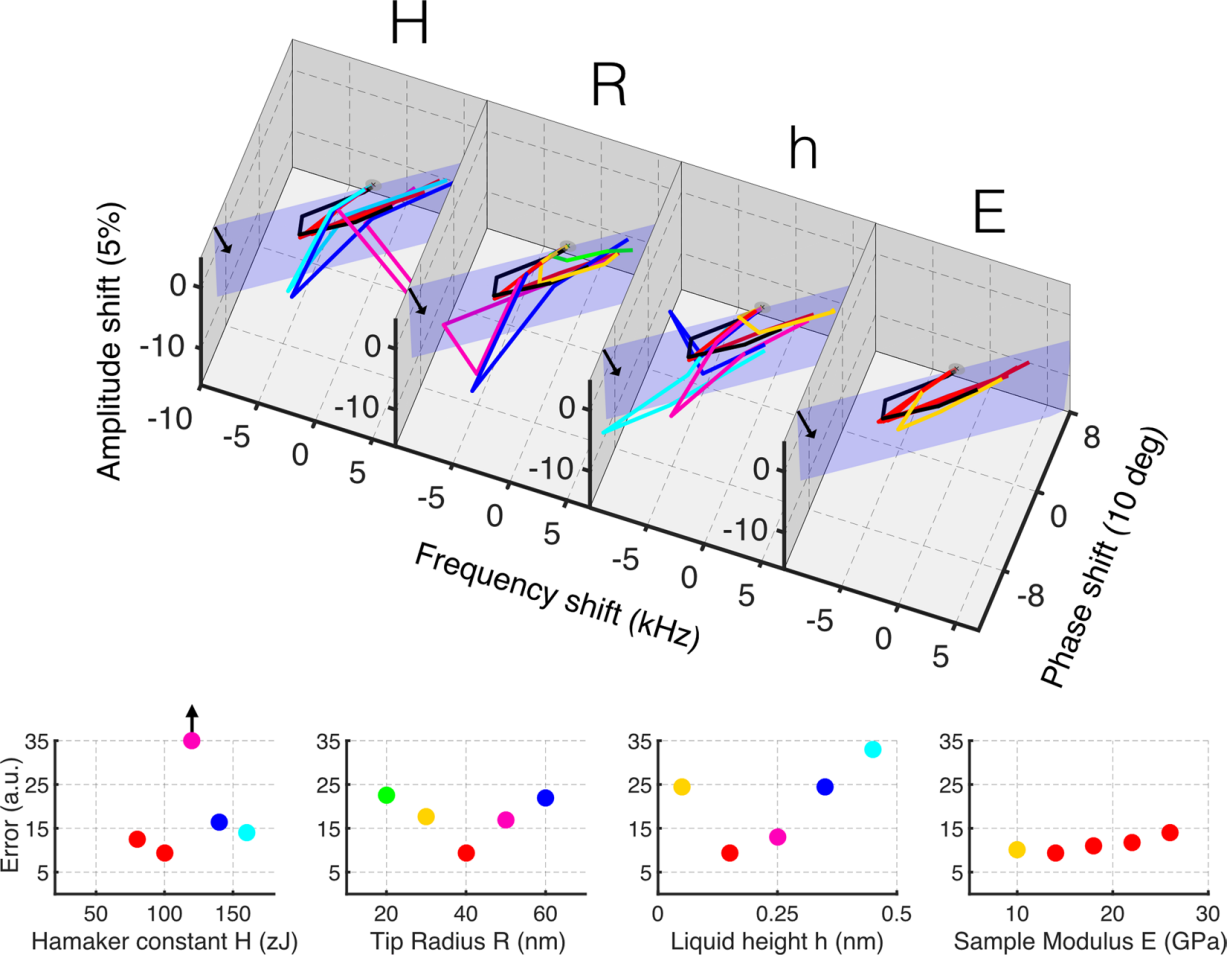
Illustration of the sensitivity of the few cycle regime in discriminating values of relevant physical parameters: Hamaker constant (H), tip radius (R), liquid height above the surface (h) and sample’s Young’s modulus (E). To illustrate the sensitivity of the method we show the experimental trajectory in black (the same as in Fig. 3) and simulated trajectories in colors (details of simulations in Methods Section) where we systematically vary one fitting parameters at a time (H, R, h or E). An optimal value for each fitting parameter minimizes the distance between the simulated trajectories and the experimental trajectory. Warm colors (yellow and green) are used for simulated metric trajectories with fitting parameters lower than optimal. Cool colors (fuchsia, blue and cyan) are used for the simulated metric trajectories with fitting parameters higher than optimal. For simplicity, all traces that are relatively close to the experimental metric trajectory are shown in red. The specific values of the physical parameters being varied are shown in the lower plots, along with the calculated error. The calculated error is the sum of the Euclidean distances between the points of the simulated metric trajectory and the corresponding points in the experimental one. The color code in these error plots is consistent with the one used in the upper plot. From these error plots we can estimate a value of Hamaker constant of 100 zJ, a tip radius of 40 nm, a water layer height of 0.15 nm and a value of sample Young’s modulus of 14 GPa. All simulations and experiments were performed at a cantilever rest position of 7.5 nm. The sinc excitation amplitudes for the points along an experimental (and simulated) metric trajectory were linearly increased as in Fig. 3.

Figure 4 reports on the sensitivity of the few cycle regime approach to the relevant physical parameters that determine the interaction dynamics, the Hamaker constant, the tip radius, the water layer height and the sample Young’s modulus. This type of analysis investigates how a variation of the physical parameters affects the metric trajectories, which ultimately dictates the sensitivity of the method. The relative error is calculated by the summation of the Euclidean distances between points in the simulated trajectories and the respective points in the experimental trajectory. It is important to note that the calculated error, which gives a quantitative estimate of the distance between a simulated trajectory and the experimental one, is not the only criterion to establish the correctness of a simulation. In fact, the experimental trajectories, as already noted, lie quasi-exactly on a plane. The simulation made using non-optimal parameter often tends to develop out-of-plane trajectories. Particularly, we noted that certain simulation values produced a prominent deviation from the plane. For example, see cyan line in Fig. 4 (left figure) when studying the effect of variation in Hamaker constant. We found out that this effect is caused by the well known jump to contact phenomenon, which causes extreme reductions in the oscillation amplitude, ultimately making the point under study to abruptly deviate from the plane. Moreover, only a restricted range of parameters allow the calculated trajectories to develop on a plane, as in the experimental data. This is a strong constraint over parameters range and, more generally, on the choice of models to simulate the tip dynamics.

Commenting more specifically the results reported in Fig. 4, we find that the simulations suggest a Hamaker constant value of approximately 100 zJ, which corresponds quite closely to the value between HOPG (our sample probed) and SiO_2_ (our tip’s material), derived from Lifshitz theory H = 132.0 zJ^19^ (see Fig. 4, label H).

The simulated metric trajectories suggest that the interacting tip had an effective tip radius of approximately 40 nm, with a very high sensitivity to small changes. This does not necessarily represent the actual tip geometry (the nominal tip radius of the cantilever used in the experiments is 10 nm), but an effective hydrodynamic radius that takes into account the adsorbed water layer and the forces it exerts, especially in the case of a liquid neck formation (see Fig. 4, label R).

It is especially remarkable how very small variations in Hamaker constant and tip radius affect significantly the metric trajectories.

It is interesting that simulations reveals an extreme sensitivity of the metric values to the presence of a water layer (see Fig. 4, label h). The water layer height is connected with the ambient relative humidity, since it is statistically more likely that a high water layer will be present at a high relative humidity, although the exact physical mechanism connecting the two parameters depends on the specific experiment and is not the focus of this work. As already noted, the implementation of a model considering the presence of a water layer is essential for reproducing the behavior of the experimental data. This can be explained by the fact that even a totally dry ambient could not be sufficient to promote a complete evaporation of a water layer adsorbed onto a surface. Figure 4 shows a measurement of relative error between the experimental trajectory at nominal 0 RH (Relative Humidity) with respect to the simulation trajectories with five different water heights. From this analysis, a water layer height of 0.15 nm minimizes the error and thus corresponds approximately to the experimental 0 RH case. It is interesting to note that even at low humidity levels (even at nominal 0 RH) the presence of a water layer is detected. This is specially remarkable when compared to standard techniques such as tapping mode, where it has been reported that the presence of a water layer is virtually undetectable^20^. Moreover, in the simulated trajectories it is evident that this technique is sensitive to small variations in the water layer height.

Perhaps the less sensitive physical quantity to track was the sample’s Young’s modulus as evidenced in Fig. 4, label E, where it is not clearly evident which simulated trajectory gives a better fit of the experimental data. In this case, where it is visually hard to see the best fit, the error analysis in Fig. 4(f) sheds light into the value of Young’s modulus that minimizes the error (14 GPa). This value is still reasonably close to the nominal stiffness value of HOPG (18 GPa). We ascribe this lack of sensitivity on the sample’s stiffness compared to other physical quantities, to the fact that in these studied configurations the attractive interaction forces govern more strongly the dynamics (and of course the metric trajectories) than the repulsive interaction forces. However, we have demonstrated in a previous publication^17^ that these conditions could be tuned to improve sensitivity regarding the elastic (or viscoelastic) properties of the sample if more ‘aggressive’ interacting conditions are chosen (i.e., larger amplitude of the sinc excitation force) to emphasize the regime governed by repulsive tip-sample interaction forces.

As a summary of the results in Fig. 4, for a displacement of 7.5 nm and a nominal 0 RH, the simulation parameters that best match the experimental metric curves on HOPG for all excitation intensities are: Young modulus *E* = 14 GPa, Hamaker constant *H* = 100 zJ, water layer height 0.15 nm and tip radius *R* = 40 nm. This underlines a very relevant point: this sensitive technique can give simultaneous information on various physical parameters (e.g., sample mechanical properties, environmental information and probe geometrical aspects) whereas in other standard techniques an *a priori* knowledge of the physical parameters is needed if one wants to extract a single physical parameter. This possibility of providing insight into multi-physical parameters is a very distinct sensitive property that we attribute to the few-cycle regime. This regime is normally overlooked by other techniques but here is successfully exploited through a sophisticated wavelet analysis.

**Figure 5:**
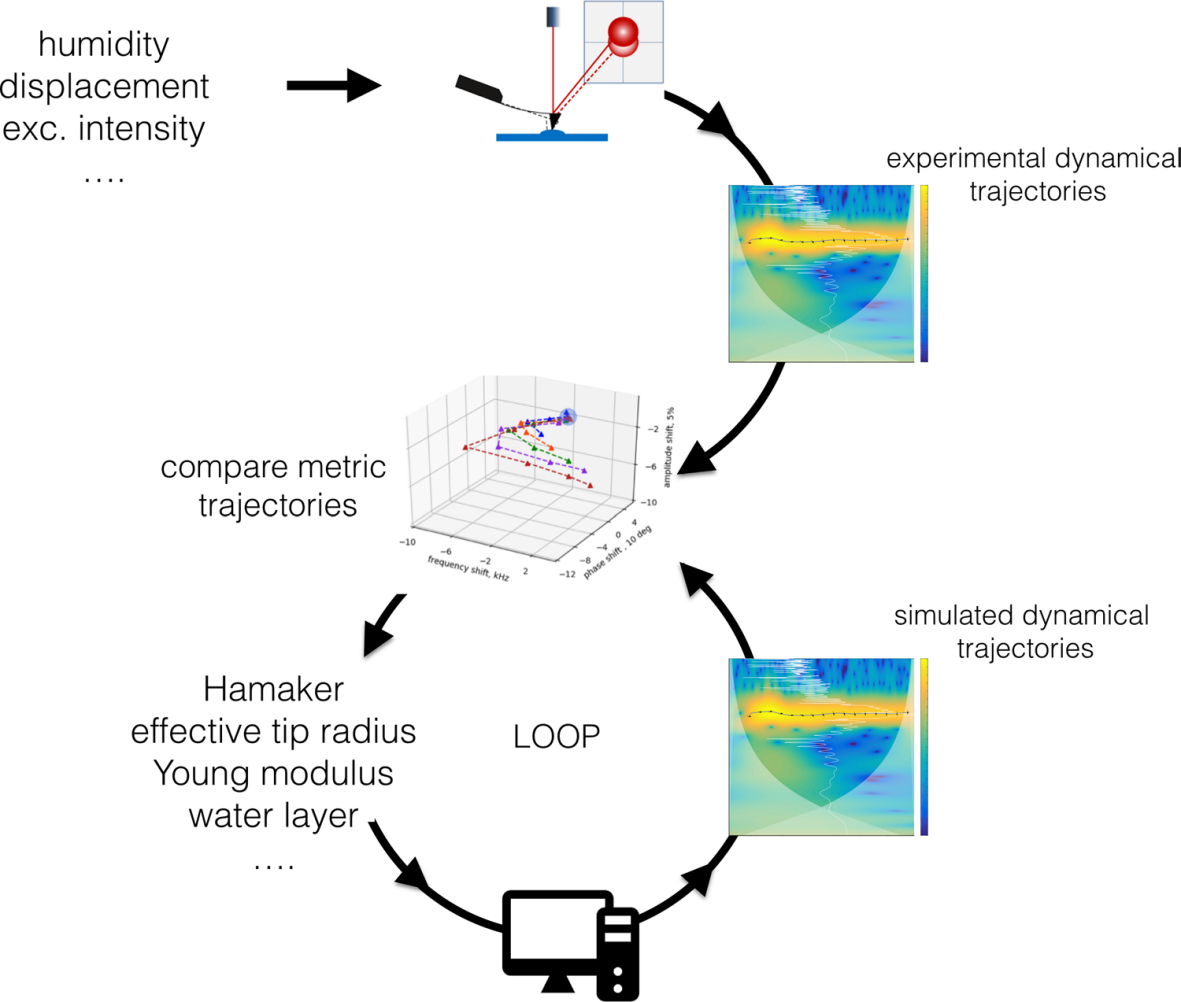
Schematics of the iterative process performed to retrieve multiple physical parameters from the experimental results. Briefly, in a first step the experimental cantilever traces with varying excitation intensities are recorded for the cases of interacting cantilever and free cantilever, followed by the corresponding calculation of the wavelet transform. Then, a *metric trajectory* is generated as described in Fig. 3. In a parallel manner a simultaneous process generating simulated *metric trajectories* is performed by systematically varying different physical parameters (e.g., water layer height, sample elastic modulus, tip radius, Hamaker’s constant) until a good fit is obtained between the *experimental* and *simulated metric trajectories* based on a minimization of error, as described in Fig. 4. As a result of this iterative process, multi-physical parameters pertaining the tip-sample contact interaction are retrieved with high accuracy.

We note that in this study we have found the ‘right’ physical parameters by systematically tuning them in the simulations (within a range where they are physically sound) in an individual fashion (while keeping the rest of the parameters constant) until replicating the experimental metric trajectories in a process summarized in the schematics of Fig. 5. When performed manually, this process of minimizing the error (to replicate the experimental metric trajectories) is time consuming and not adapted to a rapid data analysis. We envision that a more efficient algorithm including processes as trained neural networks could help in finding combinations of parameters that minimize the error in a more efficient and automated way. Despite its importance, the design of such algorithm is beyond the scope of this study and will be considered in future work. Lastly, since this technique is based on the analysis of few oscillations (unlike standard techniques based in several oscillations in the steady-state regime) it should be more susceptible to the Brownian noise of the tip present in every AFM experiment. Despite this fact, we have seen that the experimental data can be successfully processed with the wavelet analysis. We have also shown before that this does not represent a serious threat in the analysis, even for a single impact regime in highly damped environments^17^.

## Conclusions

We have presented a sensitive technique that shows promising advantages in the simultaneous characterization of multiple physical parameters, a trend recently emerged in other AFM applications^21^. Specifically, we have shown the feasibility of determining simultaneously the Hamaker constant, height of water layer adsorbed in the substrate, tip radius and sample’s elastic modulus. These results are very remarkable with regards to standard AFM techniques that work in the steady-state regime and are based in Fourier analysis. These standard AFM techniques are often either insensitive to some of these parameters or require strict *a priori* knowledge (or imposed assumptions) of some of them in order to characterize a specific physical value. We attribute the remarkable gain of sensitivity to the exploitation of a small portion of the (normally overlooked) transient regime of the tip-sample interactions (i.e., the few cycle regime), which is analyzed through a powerful mathematical tool: the wavelet transform. This tool, unlike the traditional analysis used in AFM (the Fourier analysis), allows us to track with great detail the spectral time evolution of the cantilever trajectory in a regime that encodes extremely sensitive physical information about the tip-sample interactions. Ultimately, this analysis allows us to compress the large experimental data into a set of meaningful *metric values*: amplitude shift, frequency shift and phase lag. We have shown that specific physical values (e.g., tip radius, Hamaker constant) can be retrieved by comparing these metric values of the experimental traces with those of a set of simulated traces. These sets of simulated traces are generated by systematically varying physical parameters until we are able to match the metric values of the simulation with those of the experiment. This process, if automatized in clever ways (perhaps with the aid of trained neural networks or other machine learning tools), could ensure a more efficient and successful fit (a task well beyond the scope of this paper).

In this work, we have focused our efforts in characterizing the few-cycle regime in an experiment where the cantilever is excited with a pulse-like signal. We plan to implement this concept in future work in a pixel by pixel manner to gain detailed knowledge about sample’s multi-physical parameters with the high spatial resolution offered by the AFM. In particular, these methods will be useful in characterizing mono-elemental thin films and nanosheets deposited on substrates due to their high surface sensitivity. We envision that the general concept demonstrated here will open the possibility of exploring quantitative physical parameters with a temporal resolution well beyond current AFM methods.

## Methods

### Numerical simulations

To simulate the dynamics of the cantilever interacting with the sample in an impulsive manner, we have employed a set of three ordinary differential equations coupled through the tip-sample interactions. These differential equations correspond to the dynamics of the three lower flexural eigenmodes of the cantilever (contribution of eigenmodes higher than the third are neglected).

1 $$m{\ddot{z}}_{i}(t)+\frac{m{\omega }_{0i}}{{Q}_{i}}{\dot{z}}_{i}(t)+{k}_{i}{z}_{i}(t)=f(t)+{F}_{ts}(d,{sgn}(\dot{z}))$$

*m* is the effective mass of the cantilever, *z*_*i*_, *k*_*i*_, ${\omega }_{0i}=2\pi {f}_{{0}_{i}}$, and *Q*_*i*_ refer to the *i*_*th*_ eigenmode’s (with $i=1,2,3$) displacement, force constant, resonance frequency, and quality factor, respectively. The total deflection *z*(*t*) is the sum of the individual eigenmodes’ deflections, $z(t)={\sum }_{i}\,{z}_{i}(t)$. *F*_*ts*_ represents the tip-sample forces which according to the following model depends on the tip-sample distance (*d*) and whether tip is approaching or retracting from the sample ($sgn(\dot{z})$).

The excitation force applied to the tip *f*(*t*) corresponds to the inverse Fourier Transform of the band excitation signal defined in the frequency domain^11,12^. Specifically, in the time domain the analytical transformed signal *f*(*t*) is:

2 $$f(t)=A\frac{\sin (\pi BW(t-{t}_{0}))}{\pi BW(t-{t}_{0})}\,\cos (2\pi {f}_{0}t),$$

where *A*, *t*_0_, *f*_0_, are the amplitude of the sinc excitation, the centering of the signal in time, and the centered frequency of the bandwith chosen (*BW*).

For the tip-sample forces we have employed Derjaguin-Muller-Toporov (DMT) theory^22^, with an additional term corresponding to attractive capillary forces when a thin water film of thickness *h* covers both tip and sample. These capillary forces are accounted for with a simple model described by Zitzler *et al*.^20^, where they calculate some critical distances at which the water meniscus is formed during tip approach (*d*_*on*_) and broken during tip retraction (*d*_*off*_). During approach it is assumed that meniscus is formed upon geometrical contact of the adsorbed water layers (${d}_{on}=2h$), and during retraction the meniscus is broken at a distance ${d}_{off} > {d}_{on}$ that we obtained following Willet *et al*.^23^ and Zitzler *et al*.^20^ calculations.

In this model, the tip only experiences a long range component (van der Waals interactions) when the tip is farther than the critical distances, *d*_*on*_ (during approach, $\dot{z} < 0$), and *d*_*off*_ (during retraction, $\dot{z} > 0$),

3 $${F}_{ts}=-\,\frac{HR}{6{d}^{2}};d > {d}_{on}(\dot{z} < 0),d > {d}_{off}(\dot{z} > 0)$$

*H*, and *R* are the Hamaker constant and tip radius, respectively.

Beyond meniscus formation tip-sample distance, the capillary force is added to the van der Waals interaction. This again is bounded by the critical distances *d*_*on*_ and *d*_*off*_, whether the tip is approaching or retracting, respectively.

4 $${F}_{ts}=-\,\frac{HR}{6{d}^{2}}-\frac{4\pi {\gamma }_{{H}_{2}O}R}{1+d/h};{a}_{0} < d\le {d}_{on}(\dot{z} < 0),{a}_{0} < d\le {d}_{off}(\dot{z} > 0)$$

${\gamma }_{{H}_{2}O}$ is the liquid-vapor interfacial energy of water.

Furthermore, when the approaching tip enters the repulsive tip-sample interaction regime ($d < {a}_{0}$), the Hertzian repulsive term is added,

5 $${F}_{ts}=-\,\frac{HR}{6{a}_{0}^{2}}-\frac{4\pi {\gamma }_{{H}_{2}O}R}{1+d/h}+4/3{E}^{\ast }\sqrt{R}{({a}_{0}-d)}^{3/2},d\le {a}_{0}$$

where E* is the effective Young’s modulus of the contact, defined in terms of tip and sample elastic constants,

6 $$\frac{1}{{E}^{\ast }}=\frac{1-{\nu }_{tip}^{2}}{{E}_{tip}}+\frac{1-{\nu }_{sample}^{2}}{{E}_{sample}}$$

*E*_*tip*_, *E*_*sample*_, *ν*_*tip*_, *ν*_*sample*_ are the Young’s modulus of tip and sample, and the Poisson’s ratio of tip and sample, respectively.

## Supplementary information

Supporting Info for few cycle AFM
