# Supplementary material for: Few-cycle Regime Atomic Force Microscopy
Source: Sci Rep. 2019 Sep 3;9:12721. doi: 10.1038/s41598-019-49104-1 (PMC6722071; doi:10.1038/s41598-019-49104-1)
Supplement: Supplementary file 1 — Supporting Info for few cycle AFM [file 41598_2019_49104_MOESM1_ESM.pdf]

Supporting Information for: Few cycle regime Atomic Force Microscopy

Enrique A. López-Guerra[1,2], Suhas Somnath[3], Santiago D. Solares[1], Stephen Jesse[4,5], Gabriele Ferrini[6,7]

[1] Department of Mechanical and Aerospace Engineering, The George Washington University, Washington, DC, 20052, USA.

[2] Department of Civil and Environmental Engineering, The George Washington University, Washington, DC, 20052, USA.

[3] Center for Computational Sciences, Oak Ridge National Laboratory, Oak Ridge, Tennessee 37831, USA

[4] Center for Nanophase Materials Sciences, Oak Ridge National Laboratory, Oak Ridge, Tennessee 37831, USA

[5] Institute for Functional Imaging of Materials, Oak Ridge National Laboratory, Oak Ridge, Tennessee 37831, USA

[6]Interdisciplinary Laboratories for Advanced Materials Physics, Universita Cattolica del Sacro Cuore, I-25121 Brescia, Italy

[7]Dipartimento di Matematica e Fisica, Universita Cattolica del Sacro Cuore, I-25121 Brescia, Italy

E-mail: gabriele.ferrini@unicatt.it

Wavelet Transforms

The wavelet transform (WT) is a mathematical tool well-suited for studying the spectral composition of non-stationary signals. The W represents a 1-dimensional time signal into a 2-dimensional time-frequency signal by projecting the original signal onto a base formed by wavelets. These wavelets, $\Psi_{s,u}(t)$, are dilations and translations of a mother wavelet, $\Psi(t)$, which is a smooth function with compact support and zero average ($\int_{-\infty}^{+\infty} \psi(t)dt = 0$):

$$\Psi_{s,u}(t) = \frac{1}{\sqrt{s}} \Psi\left(\frac{t-u}{s}\right) \tag{1}$$

where *s* is a scaling factor that produces dilations of the mother wavelet and *u* is a translation in time. This dilated and translated version of the mother wavelet is often referred as the daughter wavelet. The scaling *s* makes the daughter wavelet to adapt to the study of different frequency components. The variable *u* produces a time translation of the wavelet to perform the time-localized analysis of the frequency components of the studied signal.

The wavelet transform of a time-varying signal, *h(t)*, is defined as the inner product of the analyzed signal with the daughter wavelet:

$$W^h(s,u) = \langle h, \Psi_{s,u} \rangle = \int_{-\infty}^{+\infty} h\,(t) \frac{1}{\sqrt{s}} \Psi^* \left(\frac{t-u}{s} \right) dt \tag{2}$$

By Parseval's identity, the wavelet transform can alternatively be defined in Fourier space as:

$$W^h(s,u) = \frac{1}{2\pi} \langle \hat{h}, \hat{\Psi}_{s,u} \rangle = \frac{1}{2\pi} \int_{-\infty}^{+\infty} \hat{h}\,(\omega) \sqrt{s}\, \hat{\Psi}^*\,(s\omega) e^{i\omega u} d\omega \tag{3}$$

where $\omega = 2\pi f$ is the angular frequency and f the linear frequency, $\hat{h}\,(\omega)$, $\hat{\Psi}\,(\omega)$ are the Fourier transforms of $h(t)$ and $\Psi(t)$, respectively. The WT coefficients $W^h(s,u)$ are 'resemblance' coefficients that measure the similitude between the original signal, *h(t)*, with the daughter wavelets at various scales and delays[1]. Since each scale 's' can be associated to a specific frequency (as described below) and the delay 'u' is a time variable, it is now evident that the WT expresses a 1-dimentional signal (*h(t)*) into a 2-dimensional signal ($W^h(s,u)$) which describes the temporal evolution of the spectral content of the signal.

The scale s can be associated to a specific Fourier frequency for a particular wavelet function[2]. In our case, where we use as a mother wavelet the Gabor form $\Psi(t) = \frac{1}{(\pi)^{1/4}} \exp(-\frac{t^2}{2} + i\omega_0 t)$, the scale-frequency relation in the WT is to a very good approximation[3]: $f = \frac{\omega_0}{2\pi s}$, where $\omega_0$is the carrier frequency.

Wavelet Cross Correlation

A fundamental operation on the WT is the cross-correlation of two functions $h(t)$ and $r(t)$ [4]. Cross-correlation XWT is obtained by multiplying $W^h(s,u)$ by the complex conjugate transform of $r(t)$, $W^{hr} = W^h(s,u) W^r(s,u)^* = |W^h(s,u)||W^r(s,u)| \exp(i\Phi^h(s,u) - i\Phi^r(s,u))$. From the cross correlation it is possible to retrieve the instantaneous phase difference between two functions at every point (s,u). If the function $h(t)$ is seen as a driver (or cause) and $r(t)$ as a response (or consequence), from the cross-correlation it is possible to calculate the instantaneous phase shift between driver and response.

In our case, to investigate the correlation between the impulsive driver and the cantilever response we take advantage of this phase retrieved by the cross-correlation technique. To study the effect in the

dynamics caused by the tip-sample interactions we perform the XWT operation between the WT of the ‘free response’ of the cantilever to an impulsive excitation and the WT of the ‘interacting’ trajectory of the tip when subject to the same impulsive excitation. In this case we regard the ‘free response’ as a reference and the ‘interacting trajectory’ as a response to some perturbations that modify the reference signal.

By performing this operation (XWT) it is possible to calculate the instantaneous phase shift between response and reference at all frequencies. We center our attention on the phases calculated over the ‘instantaneous frequency’ trajectory. This instantaneous frequency’ trajectory can be calculated from the wavelet ridges of the normalized scalogram. Technically, the wavelet ridges are calculated from the normalized scalogram by finding the frequency points where the scalogram is maximum at a given time[5].

1. G. Malegori and G. Ferrini, *Beilstein journal of nanotechnology*, 2010, **1**, 172-181.
2. S. D. Meyers, B. G. Kelly and J. J. O'Brien, *Monthly weather review*, 1993, **121**, 2858-2866.
3. E. A. López-Guerra, F. Banfi, S. D. Solares and G. Ferrini, *Scientific reports*, 2018, **8**, 7534.
4. F. Banfi and G. Ferrini, *Beilstein J Nanotechnol*, 2012, **3**, 294-300.
5. S. Mallat, *A wavelet tour of signal processing*, Elsevier, 1999.
